# Severe Acute Respiratory Syndrome Coronavirus-2 (SARS-CoV-2): An Update

**DOI:** 10.7759/cureus.7423

**Published:** 2020-03-26

**Authors:** Mahendra Pal, Gemechu Berhanu, Chaltu Desalegn, Venkataramana Kandi

**Affiliations:** 1 Veterinary and Public Health, Narayan Consultancy on Veterinary Public Health and Microbiology, Anand, IND; 2 Epidemiology and Public Health, College of Agriculture and Veterinary Medicine, Dambi Dollo University, Dambi Dollo, ETH; 3 Epidemiology and Public Health, College of Agriculture and Veterinary Sciences, Ambo University, Ambo, ETH; 4 Clinical Microbiology, Prathima Institute of Medical Sciences, Karimnagar, IND

**Keywords:** coronavirus, beta coronavirus, respiratory and gastrointestinal infections, severe acute respiratory syndrome coronavirus-2, bats, humans and animals, aerosols, quarantine, public health, sars-cov-2

## Abstract

Coronaviruses (CoVs) belong to the family of Coronaviridae, the order Nidovirales, and the genus Coronavirus. They are the largest group of viruses causing respiratory and gastrointestinal infections. Morphologically, CoVs are enveloped viruses containing a non-segmented positive-sense, single-stranded ribonucleic acid (RNA) viruses. CoVs are categorized into four important genera that include Alphacoronavirus, Betacoronavirus, Gammacoronavirus, and Deltacoronavirus. A novel member of human CoV that has recently emerged in Wuhan, China, is now formally named as SARS-CoV-2 (severe acute respiratory syndrome coronavirus 2). This is a unique strain of RNA viruses that have not been previously observed in humans. The virus has wide host adaptability and is capable of causing severe diseases in humans, masked palm civets, mice, dogs, cats, camels, pigs, chickens, and bats. The SARS-CoV-2 typically causes respiratory and gastrointestinal sickness in both humans and animals. It can be transmitted through aerosols and direct/indirect contact, as well as during medical cases and laboratory sample handling. Specific structural proteins, which might be found on the surface of the virus, play an important role in the pathogenesis and development of the complications. The disease is characterized by distinct medical signs and symptoms that include high fever, chills, cough, and shortness of breath or difficulty in breathing. The infected people may also present with other symptoms such as diarrhea, myalgia, fatigue, expectoration, and hemoptysis. It is important from the public health and economic point of view as it affects the growth of the country, which is majorly attributed to the restriction in the movement of the people and the cost associated with the control and prevention of the disease. Since there is no specific therapeutic intervention nor a vaccine available against the virus, supportive management and treatment with non-specific therapeutic agents (repurposed drugs) may provide relief to the patients. Some preventive strategies of the disease include blocking the routes of transmission of the infections, disinfection of instruments used during medical case handling, using personal protective equipment, proper and early diagnosis of the disease, avoiding contact with the sick patients, and quarantine of the infected/exposed people.

## Introduction and background

The coronaviruses (CoVs) belong to the genus Coronavirus, the family Coronaviridae, and the order Nidovirales [1]. They are enveloped and have a non-segmented, single-stranded, positive-sense ribonucleic acid (ssRNA+) as their nuclear material. On electron microscopy, these viruses show a characteristic appearance that resembles a crown (corona in Latin means crown) due to the presence of club-shaped surface protein projections [2-3]. The CoVs are pleomorphic, measure between 80 and 160 nm in length, and have a small genome measuring 27-32 Kilobytes (KB) with a unique replication strategy [4].

**Figure 1:**
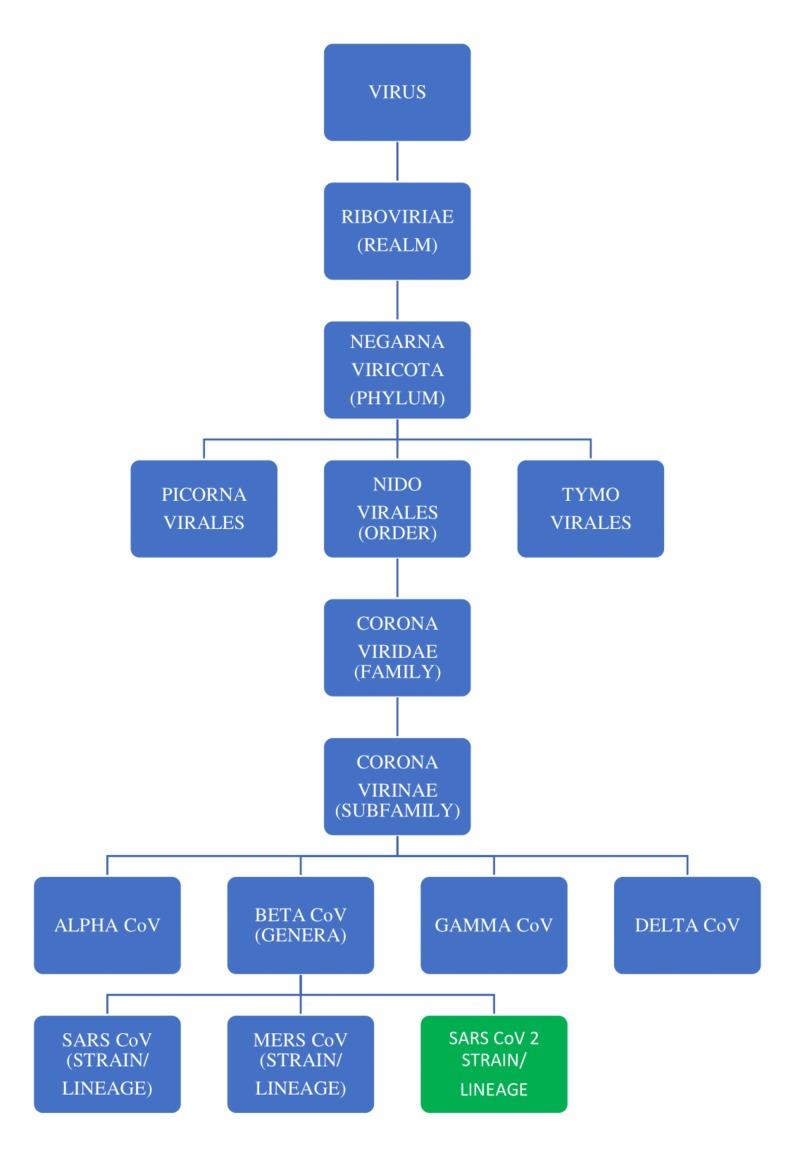
The classification of the RNA group of viruses and the origin of SARS CoV-2 CoV: coronavirus; SARS CoV 2: severe acute respiratory syndrome coronavirus 2; MERS CoV, Middle East respiratory syndrome coronavirus; RNA, ribonucleic acid

The RNA group of viruses is classified into three orders that include the order Nidovirales, which is further classified into four families: the Coronaviridae, Arteriviridae, Mesoniviridae, and Roniviridae. The family Coronaviridae is further divided into two subfamilies: Coronavirinae and Torovirinae. The Coronavirinae subfamily includes four genera of viruses (Alphacoronaviruses, Betacoronaviruses, Gammacoronaviruses, and Deltacoronaviruses), which have been grouped primarily based on serology and phylogenetic clustering (divisions based on the habitat/genetic relatedness) [5]. The detailed classification along with the origin of severe acute respiratory syndrome associated coronavirus 2 (SARS-CoV-2) is depicted in Figure 1.

A novel member of CoV capable of infecting humans was recently identified in Wuhan, China. This virus is now formally named as SARS-CoV-2. This new virus is noted to be a unique strain of RNA viruses that have not been previously observed to infect humans according to the International Committee on Taxonomy of Viruses. According to the World Health Organization (WHO), and preliminary research results, the infection with SARS-CoV-2, which was initially called as novel coronavirus disease 2019 (nCOVID-19), could result in a human infection that presents with signs and symptoms that include fever, dry cough, dyspnea, fatigue, and lymphopenia. Occasionally, human infections may lead to complications such as pneumonia, severe acute respiratory syndrome (SARS), and even death [6-7].

In this review, we attempt to update the history, genetics, epidemiology, modes of transmission, pathogenicity, clinical features, laboratory diagnosis, public health implications, economic impact, treatment, control, and prevention of SARS-CoV-2.

## Review

History

CoVs have been described as novel respiratory tract viruses, which were observed in the samples collected from the people who presented with signs and symptoms of respiratory tract infection in 1962 [4]. The first cases of SARS were recognized to have emerged in mid-November, in the year 2002 in Guangdong Province of China. According to a 2003 WHO report, the first official report of an outbreak of peculiar pneumonia within the province had affected 305 people and caused five deaths. Around 30% of cases have been suggested to occur among health care employees who were involved in patient care. More than one-third of the early cases have been noted in food handlers (individuals who manage, kill, and sell animal origin food, or those who prepare and serve food). The previous incidence of the SARS-CoV outbreak, which started in China in the year 2002 and lasted until July 2003, had spread across the world, affecting 24 countries that included Cambodia, Hong Kong, Singapore, Hanoi, Canada, and others, recording 8,437 SARS cases and 813 deaths [8-10].

In December 2019, a group of patients with pneumonia have been confirmed to be infected with a novel CoV (nCoV), which was not previously observed in humans in Wuhan, the capital city of Hubei province of China [11-13]. The newly discovered virus, causing a similar infection (SARS-CoV), was initially named as nCoV 2019 (January 2020) and was later called COVID-19 in February 2020. The WHO and the China Bureau had announced the discovery of a new coronavirus (SARS-CoV-2), which has been isolated from the patients suffering from pneumonia. As of February 12, 2020, a total of 43,103 cases of infection and 1,018 deaths have been recorded [14]. Thousands of human infections have been confirmed in China, along with many exported cases throughout the globe [15].

SARS-CoV-2 was found to infect more human beings than either of its predecessors that include the SARS-CoV and the Middle East respiratory syndrome virus (MERS) [16]. Numerous factors have contributed to the fast spread of this virus, with the main reason being the densely populated Wuhan, which is the capital city of China’s Hubei province, with more than 11,000,000 population. Because Wuhan is a transportation hub, there was an increased chance of person-to-person transmission and the possibility of exporting cases to other places [14].

Genetic characteristics and virion structure

The CoVs belong to the order Nidovirales, family Coronaviridae, and the subfamily Coronavirinae. They are genetically categorized into four important genera: the Alphacoronavirus, Betacoronavirus, Gammacoronavirus, and Deltacoronavirus. The former two genera typically infect mammals, whereas the latter two predominantly infect birds [9,17-18].

The whole-genome sequencing and the genetic analysis studies had revealed that SARS-CoV-2 is genetically related to SARS-CoV of the 2003 outbreak [14]. SARS-CoV-2 was also found to be closely related to the genus Betacoronavirus and was noted to be a distinct clade in lineage B of the subgenus Sarbecovirus, collectively with two other bat-derived SARS-CoV-like strains [12,19]. The origin of the virus is not yet clearly understood. The latest study showed that angiotensin-converting enzyme 2 (ACE 2), a membrane exopeptidase, is the receptor used by SARS-CoV-2 to enter into the human cells, much like its predecessor (SARS-CoV) [20].

All CoV genomes are organized further with the replicase locus encoded in the 5' end and the structural proteins encoded inside the 3' end of the genome. The structural proteins include the hemagglutinin esterase (HE) (only found in some beta-CoVs), spike (S), small membrane (E), membrane (M), nucleocapsid (N) and internal (I) protein, encoded inside the 'N' gene. The nucleocapsid protein complexes with the genome RNA to form a helical capsid structure observed in the viral envelope. Trimers of the spike proteins form the peplomers embedded in the envelope, giving the virion its corona or crown-like morphology. In some CoV virions, the HE protein forms smaller spikes at the membrane. The “M” and “E” also are transmembrane proteins involved in virus assembly [9].

All viruses within the Nidovirales order have a very large genome, an uncommon feature for RNA viruses, with Coronavirinae having the largest recognized RNA genomes, containing about 30 KB of genomes. Other common capabilities within the Nidovirales order encompass: (1) a notably conserved genomic organization, with a huge replicase gene preceding structural and accent genes; (2) expression of many nonstructural genes through ribosomal frameshifting; (3) several unique or uncommon enzymatic activities encoded in the large replicase-transcriptase polyprotein; and (4) expression of downstream genes with the aid of synthesis of 3′ nested sub-genomic mRNAs. In reality, the Nidovirales order was derived from these nested 3′ mRNAs as Nido is Latin means “nest”. The principal variations in the members of the Nidovirales are within the variety, kind, and sizes of the structural proteins. These variations cause considerable changes within the structure and morphology of the nucleocapsids and virions [5].

The genome consists of a 5′ cap structure together with a 3′ poly (A) tail, permitting it to behave as an mRNA, which is ready for translation of the replicase polyproteins. The replicase gene encoding the nonstructural proteins occupy two-thirds of the genome, approximately 20 kb, instead of the structural and accent proteins, which make up only approximately 10 kb of the viral genome. The 5′ end of the genome includes a leader series and untranslated region (UTR) that consists of multiple stem-loop systems required for RNA replication and transcription. Moreover, at the start of every structural or accent gene, there are transcriptional regulatory sequences (TRSs) that might be required for the expression of each of those genes. The 3′ UTR additionally consists of RNA structures required for replication and synthesis of viral RNA. The organization of the CoV genome is 5′-leader-UTR-replicase-S (Spike)-E (Envelope)-M (Membrane)-N (Nucleocapsid)-3′ UTR-poly (A) tail with accessory genes interspersed in the structural genes on the 3′ end of the genome. The accent proteins are nearly completely non-essential for the replication in tissue cultures, but some have been proven to play a crucial role in viral pathogenesis [5,21].

CoV virions are spherical, with diameters of about 125 nm, as depicted from the available research by employing cryo-electron tomography and cryo-electron microscopy. The maximum distinguished characteristic features of CoVs are the club-shaped spike projections emanating from the surface of the virion. Those spikes are a defining characteristic of the virion and deliver them the appearance of a solar corona, prompting the name coronaviruses. In the envelope of the virion is the nucleocapsid. CoVs have helically symmetrical nucleocapsids, which are unusual among positive-sense RNA viruses, however, a way more common for negative-sense RNA viruses [5,22-23].

Epidemiology

There are seven CoV species recognized to infect human beings [12]. Among these, only MERS-CoV and SARS-CoV have been able to cause severe human disease. The rest are associated with mild respiratory ailments such as the common cold. However, they may cause serious consequences in immunocompromised individuals. At present, the medical severity of the nCoV-2019 (nCoV-19) is precisely unknown but life-threatening, and deaths have been associated with the infections [24].

They have wide host adaptability and can cause severe infections in humans, birds, livestock, masked palm civets, mice, dogs, cats, camels, pigs, chickens, and bats, wherein they typically cause respiratory and gastrointestinal sickness [5,11,14,18]. Four human CoVs (HCoVs) (HCoV 229E, NL63, OC43, and HKU1) are endemic globally and account for 10% to 30% of upper respiratory tract infections in adults. Ecologically, there are several types of CoVs, and the greatest variety was noted in bats, suggesting that bats may be natural reservoirs for a lot of these viruses. Peri-domestic mammals can also serve as intermediate hosts, facilitating recombination, mutation, and genetic variations [1,25]. SARS-CoV-2 is currently spreading to different countries throughout the world including European, American, Asian, and some African countries.

Transmission

Even though the exact mechanisms of transmission are presently uncertain, human-to-human transmission can arise, and the risk of airborne spread appears imminent [3,26]. Moreover, SARS-CoV may be transmitted from bats to palm civets or dromedary camels and thereby spill over into human beings [27-28]. Reintroduction into human beings from an animal reservoir, persistent infection in previously sick people, or the laboratory strains may cause human infections and human-to-human transmission [29].

SARS is generally transmitted through direct or indirect contact of mucous membranes (eyes, nose, or mouth) with infectious respiratory droplets or fomites. Transmission risks increase with period and proximity with the contacts/infected persons [30]. The time of survival of SARS-CoV-2 within the environment is presently unknown. Recent research showed that SARS-CoV can live up to two weeks after drying and five days at temperatures of 22-25°C and 40-50% relative humidity, with a gradual decline in the viability of the virus thereafter. The viability of SARS-CoV was found to decrease after 24 hours at 38°C and 80-90% relative humidity [31]. The virus may remain viable on distinct surfaces for 48 hours at 20°C and 40% relative humidity, even though viability reduced to 8 hours at 30°C and 80% relative humidity conditions [3,32]. This confirms the fact that at low temperature, low humidity conditions favor the virus survival in the environment.

Based on the evidence of a rapidly increasing incidence of infections and the possibility of transmission by asymptomatic carriers, SARS-CoV-2 can be transmitted effectively among humans and exhibits high potential for a pandemic [6,33-36]. Additionally, the advancement and convenience of global travel could further facilitate the worldwide spread of SARS-CoV-2 [34,37]. The possibility of feco-oral transmission of SARS-CoV-2 has public health implications, especially in areas with poor sanitation [38].

Pathogenesis

CoVs demonstrate versatile host ranges and tissue tropism [14]. The preliminary attachment of the virion to the host cell is initiated by interactions between the “S” protein and its receptor [9]. The sites of the receptor-binding domain (RBD) in the S1 vicinity of a CoV’s “S” protein vary with the virus strain. Some have the RBD at the N-terminus of S1 (MHV) and the others (SARS-CoV) have the RBD at the C-terminus of S1, as noted by the results of the recent research studies [5,39-40].

CoV proteins, structural, enzymatic, and accent proteins play key roles in the pathogenesis of the CoV disease (COVID). Structural proteins, in addition to their role in the maintenance of virion shape and morphogenesis, also contribute to the viral spread in vivo and antagonizing host cellular and immune responses. Nonstructural proteins include the small accent proteins that are not at all conserved among mouse hepatitis virus (MHV) and SARS-CoVs and the 16 conserved proteins encoded in the replicase locus, a lot of which have an enzymatic role in RNA metabolism or protein processing, which further assists the viruses in antagonizing hosts immune responses during infections [9,14].

Clinical signs

CoVs naturally cause illnesses in mammals and birds that include enteritis in cows and pigs and respiratory diseases in chickens. They may also be responsible for potentially lethal respiratory tract infections (acute and chronic) in humans [5,9]. From the available preliminary clinical data, SARS can develop in COVID in stages, consisting of acute constitutional signs and symptoms, acute viral pneumonitis, acute lung damage, or even acute respiratory distress syndrome, evolving over one to two weeks. The preliminary infection could be followed by a hyperactive immune reaction, which seems to underlie the severe manifestations of SARS [41].

The incubation period of COVID may average between two to seven days (range of one to two weeks). Clinical manifestation is characterized by systemic symptoms such as high fever, chills, cough, shortness of breath or difficulty in breathing, diarrhea, myalgia or fatigue, expectoration, and hemoptysis. In severe forms, the patients may develop pneumonia, and the case fatality rates may vary considerably. Serious complications such as heart failure, respiratory failure, and liver failure most likely occur in elderly patients [8,10,13].

Respiratory failure is the most important problem of COVID; at least half of the patients (mostly the elderly people) require supplemental oxygen during the intense phase, whereas around 20% of patients progress to acute respiratory distress syndrome requiring invasive mechanical ventilator support. In contrast, the severity is usually mild in infected young children [42]. Deaths may take place as early as day 4 and as overdue as 108 days after the onset of symptoms. Virus shedding from the respiratory tract was found to peak around day 10 and later declined. Virus excretion from the gastrointestinal tract was also noted. Immunoglobulin G (IgG) antibodies had been detected 10-15 days after the onset of symptoms, and the patient’s improvement correlated with the decrease in virus load. The severity of the disease was found to correlate with increasing age, with increased mortality (up to 50%) in patients over 60 years of age [9].

Generally, early clinical signs of COVID can be similar to other seasonal viral respiratory illnesses, thereby limiting the ability of the physicians to suspect the disease at its early stages. Respiratory symptoms frequently increase from two to seven days after the onset of infection and usually include a non-productive cough and dyspnea. More severe respiratory symptoms along with rhinorrhea and sore throat may arise, which are unusual. Patients with positive laboratory tests for SARS-CoV may show advanced radiographic changes of lung indicating pneumonia after 7-10 days of infection. Many CoV infected patients (70-90%) were noted to develop lymphopenia [6].

Laboratory diagnosis

Laboratory tests, which are currently available for the diagnosis of SARS-CoV-2 in various human clinical specimens (Sputum, throat swab, nasal secretions, feces, blood/serum/plasma), include real-time polymerase chain reaction (RT-PCR), viral cultural techniques for the isolation of virus from clinical specimens, immunological tests for the detection of antibodies and antigens such as enzyme-linked immunosorbent assay, indirect fluorescent antibody technique, rapid immunochromatographic tests, and immunofluorescence techniques [7]. Other tests that may assist in the diagnosis may include the flow-cytometry analysis for CD4+ and CD8+ T cell counts, chest radiography (pneumonia), complete blood picture (to demonstrate lymphopenia), and serum biochemistry (serum protein and others) [9-10,13-14,41,43-44].

The 10 genome sequences of SARS-CoV-2 obtained from the nine patients have been noted to be extremely identical and exhibiting more than 99.98% sequence similarity. Notably, SARS-CoV-2 was closely related (with 88% similarity) to two bat-origin SARS-like CoVs (Bat-SL-CoV), Bat-SL-CoVZC45 and Bat-SL-CoVZXC21, which were collected in 2018 from Zhoushan, eastern China. Interestingly, SARS-CoV-2 was found to be non-identical with SARS-CoV (about 79% similarity) and MERS-CoV (about 50% similarity). Phylogenetic analysis revealed that the nCoV-19 fell within the subgenus Sarbecovirus of the genus Betacoronavirus, with a relatively long branch length to its closest relatives (Bat-SL-CoVZC45 and Bat-SL-CoVZXC21) and was genetically distinct from SARS-CoV and MERS-CoV. Notably, homology modeling revealed that SARS-CoV-19 had a similar RBD structure to that of SARS-CoV despite amino acid variations at some key residues [11].

Electron microscopy, virus isolation, cloning, and sequencing studies have demonstrated that an nCoV was the etiologic agent of SARS [9]. An in-depth annotation of the newly discovered CoV (SARS-CoV-2) genome has revealed differences with SARS or SARS-like CoVs. A systematic comparison study identified 380 amino acid substitutions between these CoVs, which may have caused functional and pathogenic divergences of SARS-CoV-2 [28].

Public health importance

While most CoVs cause a mild common cold-like condition in human beings (children and adults), the emergence of the agents such as SARS and SARS-associated CoVs under the subgenus Betacoronavirus highlight the nature of adaptability and genetic variations of CoVs and their potential to cause significant/serious human illnesses. Because of their novel nature and the unavailability of specific anti-viral agents and a vaccine, isolation and quarantine of exposed/infected persons appear to be of increased significance to control and prevent the spread of the virus among the general population [9,45].

SARS-CoVs have the necessary potential to cause community and nosocomial transmission and result in severe morbidity and mortality [18]. They also contribute to zoonotic infections, which may result in epidemics and represent a huge threat to public health. Previous research had suggested that CoV infection in pregnant women may result in poor obstetric consequences, which include maternal morbidity and mortality [46-48]. As noted earlier in this review, people older than 60 years of age and those with co-morbid conditions may suffer from serious/life-threatening COVID.

Economic impact

The outbreak of SARS-CoV-2 substantially affects the economic system of an individual, society, and the country as a whole. It affects transportation within the country and throughout the globe. Outbreaks result in financial losses associated with tourism, trade, and recreational activities [42]. They may also result in gross domestic product increase. The economic loss with SARS-CoV-2 may also be attributed to the loss of life of animals from the sickness and cost of treatment for both animals and humans during the outbreak [14]. Additionally, the disease has moral or psychological, legal, and political impacts throughout the world. Many countries are currently following restricted entries of foreigners. Such hindrances greatly affect the economic development of the country due to no foreign exchange.

Treatment

At present, there is no single specific anti-viral therapy available against COVID, and the treatment is mostly supportive. The cases of 2019 nCoV (SARS-CoV-2) infection have been continuously increasing throughout the world ever since its outbreak in China. Currently, SARS-CoV-2 M protein has been used as a target, which may be inhibited by the already available and approved drugs. For this reason, the safety profile of these FDA-approved drugs is carefully documented and the efficacy of the selected few can be quickly examined against the novel virus (drug repurposing). Previous studies have demonstrated the efficacy of the available drugs against SARS-CoV-2 “P” and “L” proteins and their potential to inhibit the catalytic area and inactivate the virus [11,14].

Anti-viral treatment with interferon-alpha inhalation (50 μg two times daily), lopinavir and ritonavir (anti-retroviral drugs) (400 mg twice each day and 100 mg twice every day, respectively), and arbidol (200 mg two times every day) is recommended. Patients obtained treatment with a corticosteroid (40-80 mg/day) and gamma globulin (15-20 g/day) for three to five days while their resting respiratory rate became greater than 30 per minute, or oxygen saturation was under 93% without oxygen, or multiple pulmonary lobes showed more than 50% progression of sickness in 48 hours on imaging. Patients additionally may be treated with probiotics to relieve gastrointestinal illness. Quinolones and higher generation cephalosporins (oral and intravenous) can be administered if fever lasted for more than seven days or C-reactive protein levels were 30 mg/L or more (normal range: 0-8 mg/L). After the course of treatment, the patients infected with SARS-CoV-2 can be discharged from the hospital only after two negative results of the samples collected with 24-hour interval using an RT-PCR [13]. Other drugs like chloroquine (anti-malaria drug), hydroxychloroquine (used to treat rheumatoid arthritis, Lupus), azithromycin (anti-bacterial drug), and favipiravir are all either drugs recommended for other causes (repurposed) or drugs that are under clinical trials to treat serious infections caused by SARS-CoV-2. Protease inhibitors (lopinavir/ritonavir) in combination with ribavirin may be used for anti-viral therapy in the early phase, and nelfinavir was found to be a promising alternative. The role of interferon and systemic corticosteroid therapy in preventing immune-mediated lung injury requires further investigation. Besides, other anti-viral treatments, RNA interference, monoclonal antibody, synthetic peptides, and vaccines are being developed [42]. Corticosteroids can be used to limit excessive lung damage due to an inflammatory response, and a high flow of oxygen supplementation and mechanical ventilation can be used in cases of respiratory failure [41]. Tracheostomy may be performed in patients who require prolonged mechanical ventilation and longer intensive care unit (ICU) stay. Strict adherence to infection control guidelines is mandatory while performing tracheostomy in the ICU or operating rooms, as well as during subsequent changes of the tracheostomy tube. Care should be taken during the treatment procedures to reduce complications and the chances of transmission [30]. The use of high-dose pulse methylprednisolone during the clinical course of a SARS outbreak was associated with clinical improvement, but randomized controlled trials are needed to ascertain its efficacy [49].

Control and preventive measures

The SARS-CoV disease may precipitate nosocomial transmission, and, therefore, it is important to enhance ordinary infection control measures in healthcare settings. Healthcare facilities must additionally ensure “respiratory hygiene/cough etiquette” strategy to restrict the nosocomial transmission of respiratory pathogens including SARS-CoV-2. To contain the spread of respiratory secretions, all people with signs and symptoms of respiratory infection, irrespective of presumed cause, have to be advised to cover the nose and mouth while coughing or sneezing. People must be advised to use tissues to contain respiratory secretions and get rid of them within the nearest waste disposal container. They have to also be sensitized about hand hygiene after contact with respiratory secretions and contaminated items and substances. Healthcare centers have to ensure the availability of materials (tissues and no-contact receptacles for used tissue disposal, provide conveniently placed dispensers for alcohol-based hand sanitizer, and provide cleaning soap and disposable towels for handwashing) for adhering to respiratory hygiene/cough etiquette in waiting areas for patients and site visitors [44].

Because of the potential survival of the virus in the environment for several days, the premises and areas doubtlessly contaminated with SARS-CoV-2 need to be wiped clean earlier than their re-use, with disinfectants containing antimicrobial agents recognized to be effective against CoVs. Even though there is a lack of particular evidence for their effectiveness against SARS-CoV-2, cleansing with water and household detergents and the use of common disinfectant products ought to be sufficient for general precautionary cleaning. Many antimicrobial agents have been examined against different CoVs. Several active substances, e.g., sodium hypochlorite (the household bleach) and ethanol, are extensively available in non-healthcare and non-laboratory settings [3].

A previous research study had proposed three prevention strategies for SARS [44]. These include the primary prevention strategies that deal with stopping transmission of infection to increase personal protection for individuals; secondary prevention focused on detecting the SARS infection as early as possible and referring suspected individuals to the quarantine or emergency room in the nearby medical center if needed; and tertiary prevention is focused on restoration and rehabilitation. The major strategies at the tertiary level of prevention include providing training and education in hospital and community facilities to maximize the use of preventive strategies.

Early recognition of the disease, rapid diagnosis, isolation, and stringent infection control measures are the keys to control this highly contagious disease. Isolation facilities, strict droplet and contact precautions (hand hygiene, gown, gloves, masks, eye protection), contact tracing, and quarantine/isolation of close contacts are all important measures in controlling the spread of the infection in the hospital and the community [42].

Effective staff education in infection control, personal protection equipment (PPE), disinfecting the environment, controlling patient transport, the accuracy and timeliness of the reporting and dissemination of data relating to SARS are important issues affecting public perception, thereby removing the fear and limiting the spread of disease [30]. Since SARS and any other novel infectious disease poses a great challenge to the healthcare community with medical, social, political, legal, and economic implications, all countries have to be prepared at different levels of the pandemic to deal with the threat [50]. Because there is no effective therapy or vaccine, the best measures to control is identification of the source of infection, early diagnosis, reporting, isolation, supportive treatments, and timely publishing epidemic information to avoid unnecessary panic.

## Conclusions

SARS-CoV-2 is a viral disease that is caused by an nCoV. Currently, it is one of the global issues ever since it had first emerged and caused the outbreak in China. It is now spreading to different countries of the world. The disease can be transmitted from person to person through aerosol droplets, direct and indirect contact, and handling clinical cases by the medical practitioner, as well as in the laboratory setting. Also, it can be transmitted from bats to humans, which confirms its zoonotic importance. COVID can present various clinical signs that include high fever, chills, cough, shortness of breath or difficulty in breathing, diarrhea, myalgia or fatigue, expectoration, and hemoptysis. It can be diagnosed by clinical findings and laboratory tests including serology, viral isolation, and molecular techniques. COVID has great public health and economic impact. Since the disease has no specific treatment, proper measures should be taken to control and prevent the spread.

As a future recommendation for the prevention of SARS-CoV-2, each country of the world should give attention to the diagnosis and prevention of the disease and have quarantine facilities where the suspected persons can be kept in isolation until the confirmation of the disease or otherwise, and all healthcare centers should have personal protective equipment during the diagnosis and identification of the disease. The governments of the respective countries of the world should give attention to the prevention of the disease by promoting or amending the laws concerning prevention strategies to combat the disease. The scientists, medical workers, and pharmaceutical organizations should work hard to prepare a vaccine for prevention and control and to discover a specific drug for the treatment of the disease. Most importantly, timely disease surveillance and preventive measures should be implemented all over the world to fight the disease globally.
